# The Hospitals Association and Patients' Contributions

**Published:** 1888-07-14

**Authors:** Gilbart Smith


### The Hospitals Association and Patients' Contributions.

Speeches by Mr. Burdett-Coutts, M.P., and Dr. Gilbart Smith.

*(Continued from p. 226.)*

Mr. Burdett-Coutts, M.P., on rising to respond, said: Gentlemen, the hour is so late that I feel I cannot detain you by any long speech in answer to the arguments which I have listened to against the proposals and the principle put forward in my paper. I have noted a great many points which have occurred to me, and which I should have referred to if I had the time. There are, however, one or two main arguments which have been used which I should like to say a few words about. In the first place, I should like to say that the words used by the gentleman who gave us some interesting personal reminiscences (Mr. Hill) when he addressed me, and referred to the Pay System as "your system," were hardly justified. So far from the Pay System being my system, it is a system which prevails over the whole of the civilized world except in England, and that is a point which is certainly worthy of consideration. I am just as good an Englishman as anybody else, but I do not see why, because a thing prevails abroad and another thing prevails in this country, the first is necessarily bad and the second is necessarily good. You must remember that in countries like America—a country which has been referred to —which has had to make itself by its own individual energy and enterprise, and by its reliance upon the resources of the individual, this system is widely prevalent, and in foreign countries it is universal, except that in foreign countries the complement of the various payments which in England I should propose should be met by charitable contributions are met by the State. I do not wish to introduce State aid into this country, but there is no question about it, it would be perfectly futile in this country to adopt so much of the foreign system as involves patients' payments without adopting that part of it which involves State aid. Now, there has been an argument put forward which was certainly used in—I hope I shall not be mistaken if I say—a somewhat selfish manner, and that is an argument put forward by the last speaker, in reference to the interests of the profession. I confess that was an element in the case which I approached with a considerable amount of diffidence, which I examined very carefully, and upon which I have not been able to come to a definite conclusion, because it seems to me it can only be judged by a comparison of the interests of the profession with the interests of the community. In the first place, I would examine the question whether it is an actual fact that pay hospitals would injure the profession. Dr. Steele said that the present system robs the labour market, but the argument of those who spoke for the profession was not that the Pay System would rob the labour market, but that it would defraud the general practitioner. But does it defraud the general practitioner of so much of his legitimate gains as the present system, which permits an unlimited number of people to come into the hospital promiscuously, and without any payment whatever? This is the part of the present hospital system which we want to alter. That seems to me to be a system which not only defrauds the hospital and defrauds the poor, but most certainly defrauds the profession of the payments which would legitimately find their way into the pockets of the profession, who give their services to the hospital. It seems to me that the arguments in regard to the profession are singularly contradictory. Mr. Walsh talks about unfair competition. Then Surgeon-Major Ince said he was ashamed of that argument; then Mr. Nixon said that doctors liked to give their services at the hospitals; and then comes the last speaker, who again raises the argument about the profession being defrauded. These arguments, put forward on behalf of the profession, are certainly difficult to reconcile. Doctors do like, I believe, to give their services free, but not to persons who can afford to pay for them. That is what you are doing in the present system, and that is what the Pay System would most effectually cure. So far as it does affect the profession at all, the introduction of the Pay System would certainly do more to pay the profession than the present system of promiscuous charity. At the same time, there is another point of view from which it must be judged. I want to ask whether it is a question which can positively be judged from the point of view of the profession, and whether it would be fair or proper or just to the community, and in the interests of the community that this question should be settled by a meeting of general practitioners called at four o'clock in the afternoon. It is a moot point whether there are not interests of the community to be considered; whether the interests of the public and of the poor are not to be considered; whether the economical stability of the hospital system, and the preservation of the hospitals of London, which are now in a precarious condition, have not to be considered, or are matters of no importance; whether to have the best and cheapest medical treatment ready to hand is not a matter of importance; whether, in fact, this question is not to be judged and settled, at least to a certain extent, from the point of view of the community, and not merely from the point of view even of a profession. I have been especially careful not to put myself in antagonism to the profession, and to put forward nothing that I can possibly avoid which would be prejudicial to the profession; but when the subject is raised, and a challenge is thrown down, I am bound to state that I do not think that the decision of a meeting of medical practitioners called at four in the afternoon would be the proper tribunal before which a question affecting the interests of the whole public community of this country should be decided. The only other point is one which I think is amply illustrated in my paper, but which has been made use of as an argument by Sir Sydney Waterlow, and which I think has been put forward with a considerable misconception of its exact bearing upon the case. Sir Sydney Waterlow said that these hospitals were founded and built for the sick poor, and that they were the gifts of charity for the purpose of charity. With regard to that argument, I would just state what I stated on the last occasion, that the London Hospital and any hospital which is situated as the London Hospital is, where it is unable to take any but poor patients, by reason of the poverty of the neighbourhood where it stands, is an essential part of the Pay System. But now I come to the point as to the pious founders and the difference between a foundation hospital and a hospital supported by public subscriptions. They are both for the poor. The legacies are left for the relief of the sick poor; the subscriptions, as I understand them, are for the sick poor; and what I want to ask is: Are these subscriptions, and the interest upon these foundations, going at the present moment to the sick poor? That is the whole point. If you can assure me that these hospitals confine their free beds to the sick poor, and exclude the people who can pay for themselves so long as there are poor to be provided for, then I answer that these hospitals are fulfilling the conditions upon which they were founded. I do most seriously question whether it is possible, when you have a condition of things like this, that in London out of a population of 4,000,000 there are 1,000,000 people who attend the hospitals, that is, one in four obtains hospital treatment, and the number of persons who pay is so small, that all the persons who can pay do pay. The state of things is this, that by continuing our present system, and by giving this promiscuous relief free of charge, we are doing exactly the same as if I or one of you were to walk out into the street and offer every fourth person who came by a suit of clothes free. It is as if a rich philanthropist were to say "I will give bread and meat to one person in every four in London." The figures are as plain as daylight, and by the present system you are absolutely giving free treatment to one-fourth of the population of London. Therefore I say, that if the argument of the pious founder and the charitable intentions of the originators of the hospitals is to go for anything, it is a necessary conclusion that the one-fourth of the population of London which receives relief free is unable to pay. I do not believe it. I do not wish, and have not the slightest possible desire to make any alteration in the present system, but I ask you as practical and business men, what are you going to do with regard to the present state of things? I do not myself think it is reasonable to suppose that when people really understand the state of things and the position in which this promiscuous system now is, that you will have an increase in your charitable subscriptions; but I think it is much more likely that you will have a large decrease in them. At the same time, let me point out, we have an enormous and annually increasing addition to London—an enormous increase of the demands upon these hospitals. The position is so serious and so pressing, if these great institutions are to be put upon a strong and solid basis for carrying out the real objects of charity, that it behoves everyone to look at it very carefully to see whether some self-supporting system of a practical character cannot be introduced into the hospitals, or whether they are to remain simply and solely institutions dependent upon charity. I feel myself somewhat strongly, and I do not believe myself that the profession, if they look at the matter calmly, would be willing for one moment that the hospital system should remain in its present precarious condition. I believe that the profession are anxious that that position should be better; that they are sufficiently proud of our hospitals, and sufficiently conscious of the great benefits they bestow upon their profession and their science, to be anxious to put the hospitals on a sound and clear basis. But I ask the profession, rather than that they should knock down and destroy these which are satisfactory, that they should suggest something. I hope that on some future occasion I shall have the opportunity of discussing the question further, and of answering in more detail objections which have been put forward. It has been a great pleasure to me to read the paper, but it would be very satisfactory to me if the paper should lead to any improvement in our hospital system. I have myself carefully avoided putting forward any detailed practical scheme. I thought that once it was laid down and established that something was necessary, the duty of preparing a new scheme would be best left to the attention of the governors of the hospitals.

Dr. Gilbart Smith: The Hospitals Association has now existed for four years, and many matters have been discussed; but I have not been present at any meeting where a matter so important and so far reaching in its effects upon London charities has been discussed as this question of the Pay System, which has been so ably brought before us by Mr. Burdett-Coutts. I think it would be unwise, looking at the greatness of the question, if we were to disperse without passing some resolution on the subject. I therefore move: "That this meeting requests the Council of the Hospitals Association to consider in what form, if any, this question of the admission of free patients can best be brought under the notice of the Metropolitan Hospital authorities in order to ensure its practical discussion."

The motion was put and carried unanimously, and the proceedings, after lasting three hours, closed with a vote of thanks to the chairman.

